# Effect of Hypoxia on the Calcium and Magnesium Content, Lipid Peroxidation Level, and Ca^2+^-ATPase Activity of Syncytiotrophoblast Plasma Membranes from Placental Explants

**DOI:** 10.1155/2014/597357

**Published:** 2014-08-07

**Authors:** Delia I. Chiarello, Reinaldo Marín, Fulgencio Proverbio, Zully Benzo, Sandy Piñero, Desirée Botana, Cilia Abad

**Affiliations:** ^1^Laboratorio de Bioenergética Celular, Centro de Biofísica y Bioquímica, Instituto Venezolano de Investigaciones Científicas (IVIC), IVIC-CBB, Apartado Postal 21827, Caracas 1020A, Venezuela; ^2^Laboratorio de Química Analítica, Centro de Química, Instituto Venezolano de Investigaciones Científicas (IVIC), Apartado Postal 21827, Caracas 1020A, Venezuela

## Abstract

In the current study the possible relationship between the Ca^2+^/Mg^2+^ ratio of human syncytiotrophoblast plasma membranes and their lipid peroxidation and Ca^2+^-ATPase activity was determined. Syncytiotrophoblast plasma membranes of placental explants cultured under hypoxia increased their lipid peroxidation and Ca^2+^ content, diminished their Ca^2+^-ATPase activity, and kept their Mg^2+^ content unchanged. Membranes preincubated with different concentrations of Ca^2+^ increased their Ca^2+^ content without changes in their Mg^2+^ content. There is a direct relationship between Ca^2+^ content and lipid peroxidation of the membranes, as well as an inverse relationship between their Ca^2+^ content and Ca^2+^-ATPase activity. On the contrary, preincubation of membranes with different concentrations of Mg^2+^ showed a higher Mg^2+^ content without changing their lipid peroxidation and Ca^2+^-ATPase activity. Explants cultured under hypoxia in the presence of 4 mM MgSO_4_ showed similar values of lipid peroxidation and Ca^2+^-ATPase activity of their membranes compared to those of explants cultured under normoxia. Increased Ca^2+^ content of the membranes by interacting with negatively charged phospholipids could result in destabilizing effects of the membrane structure, exposing hydrocarbon chains of fatty acids to the action of free radicals. Mg^2+^ might exert a stabilizing effect of the membranes, avoiding their exposure to free radicals.

## 1. Introduction

Preeclampsia is a clinical syndrome characterized by vascular endothelial damage, hypertension, proteinuria, edema, generalized arteriolar vasospasm, and a state of oxidative stress [1]. One of the primary events in the pathophysiology of preeclampsia is reduced trophoblast invasion, which results in deficient conversion of the uterine spiral arteries during placentation [2]. This event, widely accepted as a key feature in the pathophysiology of preeclampsia, leads to a reduced placental perfusion and therefore to hypoxia, which has been linked to oxidative stress [3], a condition occurring when the body's antioxidant defenses are overwhelmed by the generation of reactive oxygen species (ROS). ROS can promote lipid peroxidation and vascular endothelial damage, which are commonly seen with preeclampsia [4, 5]. The placenta is considered to be the principal source of ROS in preeclamptic women, but maternal leukocytes and endothelium are also likely contributors [6].

Interaction of ROS with lipids, proteins, and carbohydrates of the plasma membranes can increase their level of lipid peroxidation, thus decreasing their fluidity and the activity of membrane enzymes [7]. Particularly, the plasma membrane Ca^2+^-ATPase (PMCA) is dependent upon lipid-protein interactions, and its activity is greatly affected by the level of lipid peroxidation in its environment [8–12].

Incubation of placental tissue under hypoxic conditions, which occurs with preeclampsia, induces oxidative stress, the release of proinflammatory cytokines, and trophoblast cell death [13–16]. Consequently, incubation of placental villous fragments in hypoxia has been used as placental model of preeclampsia and also serves as a good source of placental lipid peroxides [17].

Interestingly, tissues maintained under periods of hypoxia increase their intracellular calcium [18], an effect that is worsened when the activity of the PMCA is decreased [17]. Higher intracellular calcium concentrations could increase the calcium content of the cell membranes, and then the ion, acting as an amplifier, could enhance their mechanisms of lipid peroxidation. It has been reported that calcium is able to alter the stability of macrophage plasma membranes, making them more sensitive to photoperoxidation by UV light [19].

Kisters and collaborators [20] observed a lower content of Mg^2+^ and a higher content of Ca^2+^ in membranes of red blood cells from preeclamptic pregnant women, as compared with the red blood cells of uncomplicated pregnant women. These alterations could lead to interactions of these ions with membrane components, resulting in modifications of the lipid microenvironment that interacts with membrane transporters. It is well known that the interaction of metal cations and lipids has a significant impact on membrane properties, such as the area per lipid or chain ordering [21].

In the current study, we incubated syncytiotrophoblast plasma membranes with different concentrations of CaCl_2_ and MgSO_4_, in order to modify their Ca^2+^/Mg^2+^ ratio. In addition, syncytiotrophoblast plasma membranes were prepared from placental explants cultured under either normoxic or hypoxic conditions, in order to evaluate possible changes in the Ca^2+^/Mg^2+^ ratio of their cell membranes. The results were used to try to establish a relationship between the Ca^2+^/Mg^2+^ ratio, the lipid peroxidation, and the PMCA activity of the syncytiotrophoblast plasma membranes.

## 2. Materials and Methods

### 2.1. Placenta Collection

Term placentas obtained from uncomplicated (normal) pregnant women were collected immediately after delivery from the “Concepción Palacios” Maternity Hospital, Caracas, Venezuela, and transported to our laboratory on ice. All the women participated in this study in accordance with the ethical standards established by the Declaration of Helsinki. The study protocol was approved by the Institutional Review Board of “Concepción Palacios” Maternity and the Bioethics Committee of IVIC and all women gave signed informed consent. Patients were followed from the time of admission and then before and after delivery. All of the pregnant women enrolled in the study were nulliparous, gave birth by vaginal delivery, belonged to urban population of Caracas, and had similar demographic backgrounds. They had no history of hypertension or proteinuria during the pregnancy and gestational age was estimated from the date of the last menstrual period and confirmed by ultrasonography. The characteristic averages of the participating pregnant women are shown in Table 1. Criteria for exclusion from the study were a history of hypertension, diabetes, Ca^2+^ metabolism disorders or any other chronic medical illness, or over 1 g of supplemental Ca^2+^ per day during the pregnancy.

### 2.2. Explant Culture

After removal of the chorionic plate and about 0.25 cm of decidua, explants were prepared using only tissue from the intermediate region of the placenta. In brief, randomly sampled villous tissue fragments, of roughly 0.5 cm × 0.5 cm, were cleaned of large vessels and blood clots, rinsed 5 times in cold sterile saline, placed in 12-well plates (Nunclon TM Surface) containing 2 mL of DMEM-F12 medium and 10% fetal calf serum as culture medium, and enriched by addition of crystalline penicillin 100,000 UI/mL, gentamicin 48 *μ*g/mL, and amphotericin B 3 *μ*g/mL. The preparation of placental explants was carried out on ice at 4°C and completed in approximately 30 min. The explants prepared in this way, without further incubations, were identified as freshly prepared placental explants.

The tissue explants were incubated for 4 h at 37°C in 4 mL medium (DMEM-F12 with 10% FCS) in a sterile CO_2_ incubator (Shel Lab Model IR2424) with a gas mixture that composed of 8 percent O_2_, +87 percent N_2_+5 percent CO_2_ (normoxia), with constant gas pressure. The culture medium was then removed and replaced with new culture medium. The explants were divided in two groups: one group was kept under normoxia for 18 h at 37°C and the other group was cultured under hypoxia (2 percent O_2_, +93 percent N_2_+5 percent CO_2_) for 18 h at 37°C. At the end of the culture period, the explants were carefully removed and rinsed 5 times in cold sterile saline. The explants were then used for the isolation of syncytiotrophoblast plasma membranes.

### 2.3. Preparation of Syncytiotrophoblast Plasma Membranes

The membranes were prepared from placental explants following a method previously described [22, 23]. In brief, the maternal decidua was removed, and the central portion between the maternal and fetal surfaces was used for the preparation. Placental villous tissue (80–100 g) was chopped into small pieces, washed with 0.9% NaCl to remove blood, and filtered through gauze. The purification method involved different steps: differential centrifugation, precipitation of nonmicrovillus membranes with magnesium ions, and a sucrose gradient step. All solutions were buffered with 20 mM Tris-maleate, pH 7.4. Sucrose gradient preparation: a portion (2-3 mL) of the microvillus-enriched preparation and the basal membrane-enriched preparation was overlaid on the sucrose gradient. The band at the sucrose interface concentrations 37/45% (w/v) corresponds to the microvillus membrane fraction (MVM) and the band at the sucrose interface concentrations 47/52% (w/v) corresponds to the basal membrane fraction (BM). The two fractions were collected, diluted 10-fold with the buffer 20 mM Tris-maleate, pH 7.4, and centrifuged at 110,000 ×g for 30 min. The plasma membranes were resuspended in 300 mM sucrose, 20 mM Tris-Maleate, pH 7.4, and stored at −70°C (freezer). The purity and enrichment of the membrane fractions were determined routinely by assaying for adenylate cyclase/*β*-adrenergic receptor (by measuring ^3^H-dihydroalprenolol binding), as well as cytochrome-c oxidase/succinate dehydrogenase and glucose-6-phosphatase and alkaline phosphatase activities [23, 24].

### 2.4. Preincubation of Syncytiotrophoblast Plasma Membranes with Ca^2+^ and Mg^2+^

Aliquots of BM and MVM (1 mL of a 1 mg protein/mL suspension) were preincubated for 24 h at 0–4°C with a solution of 150 mM NaCl and, according to the experimental design, different concentrations of Ca^2+^ (as CaCl_2_) and Mg^2+^ (as MgSO_4_), in order to modify their Ca^2+^ and Mg^2+^ content. At the end of the preincubation period, the suspension was diluted with a solution containing 250 mM sucrose and 10 mM Tris-Hepes (pH 7.2) and washed 6 times by centrifugation-suspension at 47,500 ×g in order to remove traces of Ca^2+^ and Mg^2+^. The membranes were resuspended with the same washing solution (0.8 mg protein/mL, final concentration) and kept at −70°C until use. The membranes were used to determine their PMCA activity, TBARS, and Ca^2+^ and Mg^2+^ content.

### 2.5. Determination of Ca^2+^ and Mg^2+^ Contents of Syncytiotrophoblast Plasma Membranes

Red blood cell ghosts were prepared with a modification of a previously described method [25]. Aliquots of BM and MVM were washed three times by centrifugation-resuspension at 47,500 ×g for 45 min. The membrane pellet was resuspended with a solution containing 250 mM sucrose and 10 mM Tris-Hepes (pH 7.2) and the protein concentration was adjusted to 1.5 mg/mL. A 200 *μ*L aliquot of the membrane suspension was mixed with 500 *μ*L of 70% nitric acid and placed in a heating block (Fisher Scientific Dry Bath incubator) at 80°C for 24 h. After 24 h digestion, 200 *μ*L of 60% perchloric acid was added, and the digestion was continued for 24 h at 80°C. Finally, the whole mixture was suspended with 2 mL of MQ water. The Ca^2+^ and Mg^2+^ contents of the sample were measured by inductively coupled plasma spectroscopy (Perkin Elmer Optima 3000 DV ICP system).

### 2.6. PMCA Activity

The ATPase activity was determined by measuring the quantity of inorganic phosphate liberated from the hydrolysis of ATP, following a modification of the method described elsewhere [26]. Briefly, 180 *μ*L of the incubation medium was preincubated for 2 min at 37°C, and the reaction was started by the addition of 20 *μ*L of membrane suspension. After 10 min of incubation, the reaction was stopped by the addition of 300 *μ*L of a cold solution containing 2.85% ascorbic acid, 1.76% HCl, 0.48% ammonium molybdate, and 2.85% SDS. The samples were shaken and kept at 0°C for 10 min. Then, 500 *μ*L of a solution of 2% sodium citrate, 2% sodium arsenite, and 2% glacial acetic acid was added to each tube. The tubes were shaken and then incubated for 10 min at 37°C. The absorbance of each tube was determined in a Sunrise (Tecan) spectrophotometer at 705 nm. The PMCA activity was calculated as the difference in the phosphate liberated in a medium containing 250 mM sucrose, 5 mM ATP, 5 mM MgCl_2_, 1 mM ouabain, 2 mM EGTA, 2 mM EDTA, 30 mM Tris-HCl (pH 7.2 at 37°C), 55 mM KCl, 2 *μ*g/mL calmodulin, 1 mM thapsigargin, and 2 *μ*M free calcium, minus the one liberated in the same medium, but in the absence of calcium. A blank control was run in parallel under the same conditions, except for the fact that the membrane suspension was added only after the inclusion of ascorbic acid solution. The ATPase activity was expressed as nmol Pi/mg protein per min, after subtraction of the value obtained with the blank. The protein concentrations were determined using the Bio-Rad microassay [27]. In order to avoid the presence of membrane vesicles, membrane fractions were pretreated with SDS before the assays, as previously described [28]. The optimal SDS/protein ratio was ~0.2 *μ*g SDS/*μ*g protein.

### 2.7. Lipid Peroxidation Measurements

The amount of lipid peroxidation of the plasma membranes was estimated by measuring the thiobarbituric acid-reactive substances (TBARS). The TBARS was determined according to the method described by Feix et al. [29]. The values are expressed as nmoles of malondialdehyde per mg of protein.

### 2.8. Statistical Analysis

Comparisons between the different conditions were assessed by one-way ANOVA with the post hoc analysis with the Student-Newman-Keuls test. All results are expressed as means ± standard error (S.E.) and* n* represents the number of experiments performed with different preparations. ATPase activities were calculated from paired data. A *P*  value < 0.05 was accepted as statistically significant.

## 3. Results

Level of lipid peroxidation (determined as TBARS), PMCA activity, and Ca^2+^ and Mg^2+^ content of syncytiotrophoblast plasma membranes (BM and MVM) from freshly prepared placental explants are shown in Table 2, and those from placental explants cultured under either normoxic or hypoxic conditions are shown in Table 3. While the values of all the analyzed parameters for both BM and MVM from explants cultured in normoxic conditions are quite similar to those shown in Table 2 (freshly prepared explants), the membranes prepared from explants cultured under hypoxic conditions showed an increase in lipid peroxidation and Ca^2+^ content, as well as an important diminution in PMCA activity. Hypoxia did not produce any change in the Mg^2+^ content of either BM or MVM. These results suggest that hypoxia, by increasing the Ca^2+^ content of the syncytiotrophoblast plasma membranes, stimulates lipid peroxidation, which in turn inhibits PMCA activity.

**Figure 1:**
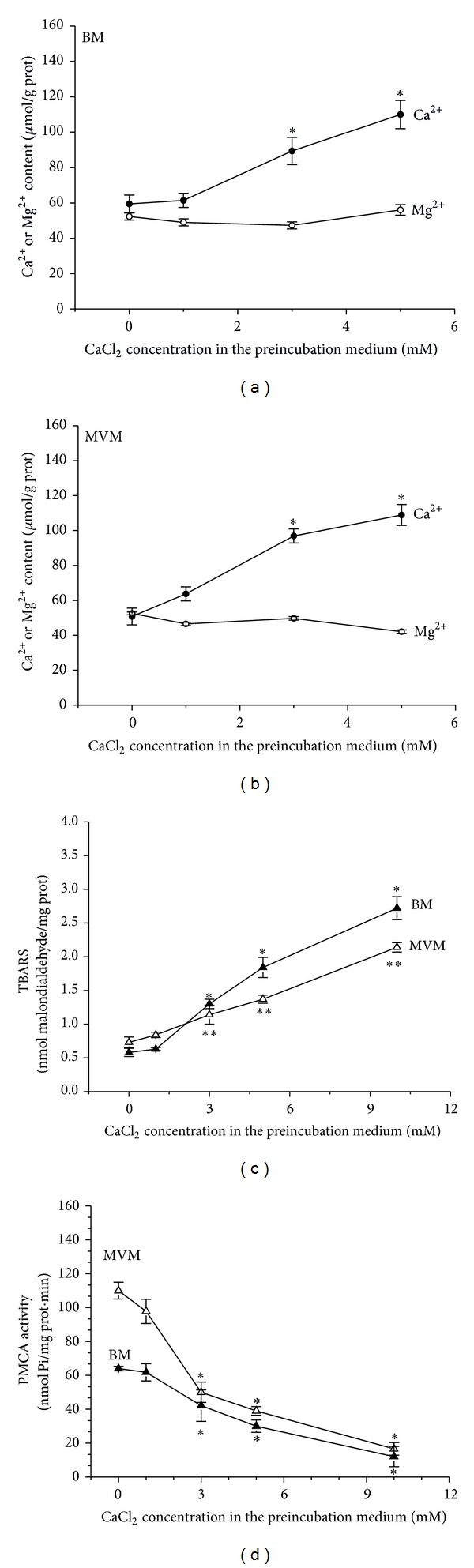
Panels (a) and (b). Ca^2+^ and Mg^2+^ content of syncytiotrophoblast plasma membranes (BM and MVM, resp.) prepared from freshly prepared placental explants and then incubated with different concentrations of Ca^2+^. Values expressed as means ± S.E., for *n* = 4. Panels (c) and (d). Level of lipid peroxidation, determined as TBARS, and PMCA activity, respectively, of syncytiotrophoblast plasma membranes (BM and MVM) prepared from freshly prepared placental explants and then incubated with different concentrations of Ca^2+^. Values expressed as means ± S.E., for *n* = 4. *P < 0.001 versus 0 CaCl_2_.

These effects of hypoxia were produced on plasma membranes prepared from placental explants cultured under hypoxic conditions with intact chorionic villi. However, the integrity of the cells might not be necessary to achieve these results. In order to rule out this possibility, BM and MVM were prepared from freshly obtained placental explants and then incubated with different concentrations of CaCl_2_, in order to try to alter their Ca^2+^ content. The membranes were then analyzed for Ca^2+^ and Mg^2+^ content, level of lipid peroxidation, and PMCA activity. The results in Figures 1(a) and 1(b) show that incubation of both BM and MVM with different concentrations of CaCl_2_ increased their Ca^2+^ content, without affecting their Mg^2+^ content. In addition, the level of lipid peroxidation of the membranes increased (Figure 1(c)) while the PMCA activity decreased (Figure 1(d)).

**Figure 2:**
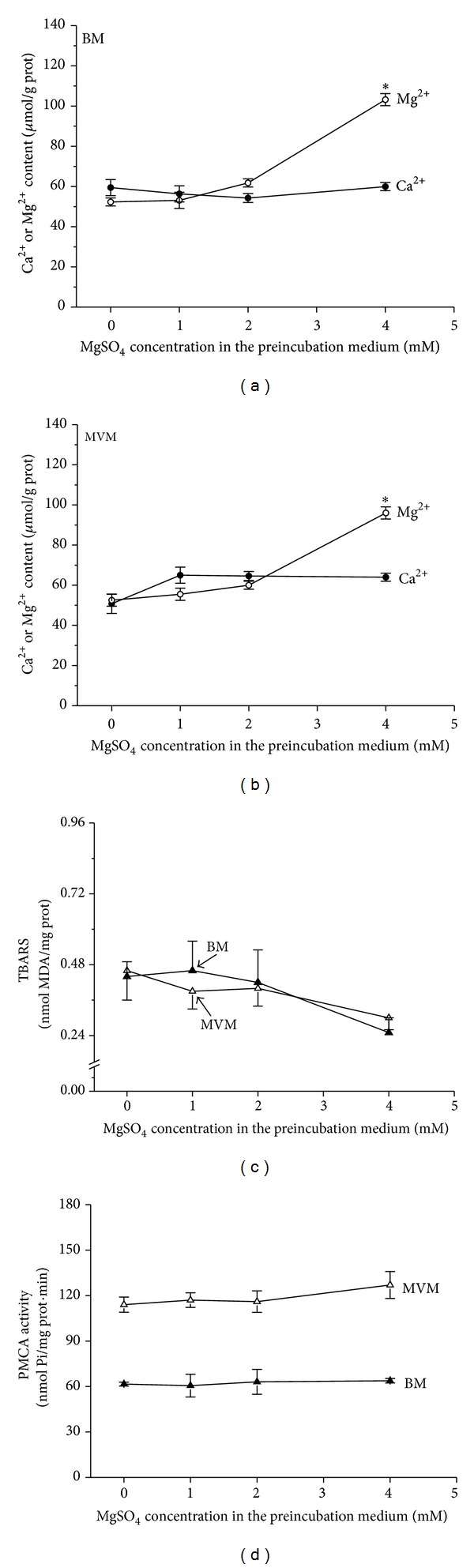
Panels (a) and (b). Ca^2+^ and Mg^2+^ content of syncytiotrophoblast plasma membranes (BM and MVM, resp.) prepared from freshly prepared placental explants and then incubated with different concentrations of Mg^2+^. Values expressed as means ± S.E., for *n* = 3. Panels (c) and (d). Level of lipid peroxidation, determined as TBARS, and PMCA activity, respectively, of syncytiotrophoblast plasma membranes (BM and MVM) prepared from freshly prepared placental explants and then incubated with different concentrations of Mg^2+^. Values expressed as means ± S.E., for *n* = 3. *P < 0.001 versus 0 MgSO_4_.

The variations shown in Figure 1 can be explained as the effect of increased Ca^2+^ on the membranes. However, as there were no changes in the Mg^2+^ content of the membranes, it is possible that the effects were due to an alteration of their Ca^2+^/Mg^2+^ ratio, rather than to just their Ca^2+^ content. In order to study this possibility, syncytiotrophoblast plasma membranes were preincubated in the presence of different concentrations of Mg^2+^ (as MgSO_4_) and then analyzed for Ca^2+^ and Mg^2+^ content, level of lipid peroxidation, and PMCA activity. The results of this experiment are shown in Figure 2.

Figures 2(a) and 2(b) indicate that while lower concentrations of MgSO_4_ in the preincubation medium had a minimal effect on the Mg^2+^ content of the syncytiotrophoblast plasma membranes, a concentration of 4 mM MgSO_4_ was able to significantly increase their Mg^2+^ content. None of the tested concentrations of MgSO_4_ altered the Ca^2+^ content of the membranes (Figures 2(a) and 2(b)), the TBARS (Figure 2(c)), or the PMCA activity (Figure 2(d)).

**Figure 3:**
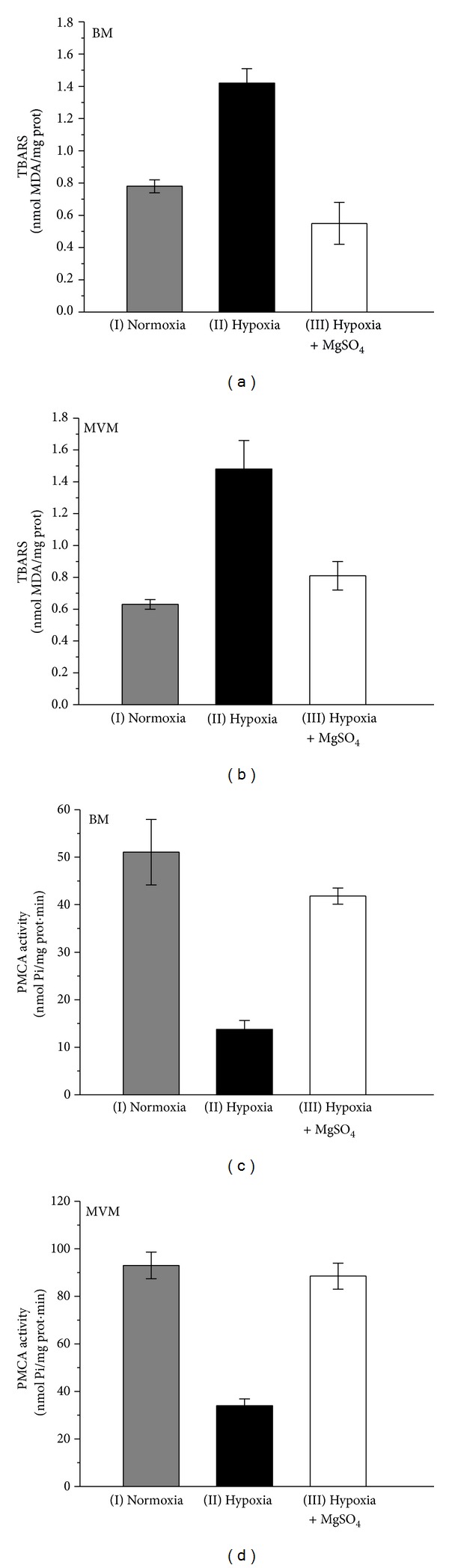
Panels (a) and (b). Lipid peroxidation (as TBARS) of basal (BM) and microvillus plasma membranes (MVM) prepared from normal human term placental explants, previously cultured under either normoxia, hypoxia, or hypoxia + 4 mM MgSO_4_. Values expressed as means ± S.E., for *n* = 5. Panels (c) and (d). PMCA activity of basal (BM) and microvillus plasma membranes (MVM) prepared from normal human term placental explants, previously cultured under either normoxia, hypoxia, or hypoxia + 4 mM MgSO_4_. Values expressed as means ± S.E., for *n* = 5. (II) versus (I) *P* < 0.001. (III) versus (I) n.s. (III) versus (II) *P* < 0.001.

If the molecular events occurring during culture of the placental explants under hypoxic conditions are similar to the response when the placental plasma membranes are incubated with Ca^2+^, then it should be possible to circumvent the rise of the Ca^2+^ content of the membranes by adding Mg^2+^ to the preincubation medium. Therefore the hypoxia experiments were repeated but with Dulbecco's Modified Eagle culture medium (1.8 mM CaCl_2_ and 0.8 mM MgSO_4_) used for incubation of the placental explants adjusted to a final concentration of MgSO_4_. The results of these experiments showed that the presence of 4 mM MgSO_4_ prevented both the lipid peroxidation of syncytiotrophoblast plasma membranes (Figures 3(a) and 3(b)) and the decrease in the PMCA activity (Figures 3(c) and 3(d)).

## 4. Discussion

In this study it was found that syncytiotrophoblast plasma membranes (BM and MVM) of placental explants cultured under hypoxic conditions showed a significant increase in Ca^2+^ content and lipid peroxidation, as well as an important diminution in PMCA activity, when compared to plasma membranes either from freshly prepared placental explants or from placental explants cultured under normoxic conditions. In these experiments, the membrane Mg^2+^ content remained constant (Tables 2 and 3). Similar results were obtained by preincubating syncytiotrophoblast plasma membranes with different concentrations of Ca^2+^ (Figure 1). The Mg^2+^ content of the membranes increased when the preincubation media contained 4 mM MgSO_4_, but it remained unchanged after preincubations with lower magnesium concentrations. However, varying the concentration of Mg^2+^ in the preincubation medium did not change the membrane Ca^2+^ content, level of lipid peroxidation, or PMCA activity.

These results may suggest that hypoxia, by increasing the Ca^2+^ content of the syncytiotrophoblast plasma membranes, stimulates their level of lipid peroxidation which, in turn, inhibits their PMCA activity. How can changes in the membrane Ca^2+^ content modify the degree of lipid peroxidation? Cations have been proposed to penetrate deeply into the head group region of the membrane, probably binding between the carbonyl oxygen and the phosphate group [30]. Consequently, there is the possibility of a direct interaction of calcium with negatively charged phospholipids of the cell membrane. This interaction could result in a destabilizing effect on the structure of the membrane, producing changes in the orientation of their phospholipids that expose hydrocarbon chains of fatty acids to the action of free radicals, thus making them more susceptible to peroxidation. It is widely accepted that Ca^2+^ ions interact with plasma membranes to produce important changes in their lipid profile; that is, phospholipid scrambling in the human erythrocyte membrane involves the synergistic action of phosphatidylinositol 4,5-bisphosphate and Ca^2+^ [31].

A relationship between lipid peroxidation and PMCA activity has been established for different cell membranes [9–12, 32]. Membrane ATPases have a strong dependence on lipid-protein interactions and consequently their activities are greatly affected by the degree of lipid peroxidation in the surrounding microenvironment [33]. Consistent with this, we found that with the increased level of lipid peroxidation there was diminution of the PMCA activity of syncytiotrophoblast plasma membranes (Table 3 and Figure 1). These results could be relevant for understanding the molecular basis of preeclampsia, as it has been found that red blood cell ghosts from preeclamptic women show high levels of membrane Ca^2+^ and low levels of membrane Mg^2+^ [20] and also high levels of lipid peroxidation and low PMCA activity [34].

Parenteral MgSO_4_ administration is the medical treatment for preeclamptic women to prevent the recurrent seizures of eclampsia and for tocolysis in preterm labor [35]. In previous experiments, we have found that, twenty-four hours after the onset of MgSO_4_ therapy, both the level of lipid peroxidation and PMCA activity of red blood cell membranes from preeclamptic women show values similar to those of normotensive pregnant women [34]. A similar effect was also seen when red blood cells from untreated preeclamptic women were incubated* in vitro* with 4 mM MgSO_4_ for 24 h at 0°C [34]. In addition, the results shown in Figures 2(a) and 2(b) indicate that incubation of syncytiotrophoblast plasma membranes with sufficient MgSO_4_ increases their Mg^2+^ content. Taken together, it may be concluded that the MgSO_4_ treatment raises the Mg^2+^ content of the red cell ghosts and thereby reduces the level of lipid peroxidation and increases the PMCA activity of these membranes. Furthermore, the results presented in Table 3 indicate that BM and MVM prepared from explants cultured under hypoxia in 1.8 mM CaCl_2_ and 0.8 mM MgSO_4_ (Dulbecco's Modified Eagle Medium) show a significant increase in their Ca^2+^ content, as compared to results from explants cultured under normoxia, with no variations in their Mg^2+^ content. However, increasing the concentration of the culture medium to 4 mM MgSO_4_ protected the membranes against the effects of hypoxia (Figure 3). These results might be an indication that a minimum concentration of Mg^2+^ is required to protect the membranes and to circumvent the rise of lipid peroxidation and the inhibition of the PMCA activity of the syncytiotrophoblast plasma membranes seen during preeclampsia [34].

The exact molecular mechanisms explaining the interactions of Ca^2+^ and Mg^2+^ within the plasma membranes of preeclamptic women remain to be determined. However, our study points out the importance of these interactions for the control of the level of lipid peroxidation of the plasma membranes and for the activity of transporting proteins such as the PMCA during preeclampsia.

## Figures and Tables

**Table 1:** Clinical data from uncomplicated pregnant women.

	Averages
Number of pregnant women	10
Maternal age (yr)	21.6 ± 3.7
Percent nulliparous	100
Mean blood pressure at delivery (mmHg)	83.5 ± 3.3
Gestational weeks at delivery	39.9 ± 0.7
Birth weight (g)	2973 ± 156
Placental weight (g)	522 ± 37
Vaginal delivery (%)	100

Data are shown as mean ± S.E. Dichotomous variables are given as a percentage.

**Table 2:** Lipid peroxidation (as TBARS), PMCA, and Ca^2+^ and Mg^2+^ content of syncytiotrophoblast plasma membranes from freshly prepared placental explants.

Measured parameters	BM	*n*	MVM	*n*
TBARS (nmol malondialdehyde/mg prot)	0.46 ± 0.11	(5)	0.61 ± 0.17	(5)
PMCA activity (nmol Pi/mg prot*·*min)	61 ± 4	(5)	110 ± 5	(5)
Membrane Ca^2+^ content (*µ*mol/g prot)	67 ± 4	(5)	75 ± 5	(8)
Membrane Mg^2+^ content (*µ*mol/g prot)	50 ± 3	(4)	55 ± 3	(8)

Values are expressed as the means ± S.E. for the indicated *n* between parentheses.

**Table 3:** Lipid peroxidation (as TBARS), PMCA, and Ca^2+^ and Mg^2+^ content of syncytiotrophoblast plasma membranes from placental explants cultured under either normoxia or hypoxia conditions.

Measured parameters	BM		MVM	
	Normoxia	Hypoxia	Normoxia	Hypoxia
TBARS nmol malondialdehyde/mg prot	0.72 ± 0.04 (4)	1.42 ± 0.09 (4)∗∗	0.63 ± 0.03 (4)	1.48 ± 0.18 (4)∗∗
PMCA activity nmol Pi/mg prot*·*min	51.0 ± 6.9 (5)	13.7 ± 1.9 (5)∗∗	93 ± 5.6 (5)	34 ± 2.9 (5)∗∗
Membrane Ca^2+^ content *µ*mol/g prot	56 ± 3 (5)	82 ± 4 (6)∗∗	66 ± 5 (6)	101 ± 8 (6)∗
Membrane Mg^2+^ content *µ*mol/g prot	54 ± 4 (6)	51 ± 2 (6)	57 ± 3 (6)	58 ± 4 (6)

Preincubations were carried out for 18 h at 37°C. Values are expressed as the means ± S.E. for the indicated *n* between parentheses.

*P < 0.01 versus normoxia.

**P < 0.001 versus normoxia.
